# Case report of zoonotic infection of *Pasteurella Multocida* leading to an early stage empyema after a right upper lobectomy

**DOI:** 10.1093/jscr/rjag043

**Published:** 2026-02-20

**Authors:** Michael Kay

**Affiliations:** Department of Cardiothoracic Surgery, Essex Cardiothoracic Center, Basildon, SS16 5NL, United Kingdom

**Keywords:** *Pasteurella Multocida*, empyema, thoracic surgery, lobectomy

## Abstract

A 77-year-old patient who underwent a right upper lobectomy and lower lobe wedge resection presented with a stage two empyema 3 days postoperatively. The presence of a zoonotic disease of *Pasteurella Multocida* was confirmed postoperatively having undergone a washout of the thoracic cavity and decortication. The patient had isolated *P. Multocida* within the pleural fibrin only and mucopurulent fluid initially mistaken for post haemorrhagic changes on computed tomography of the thorax. The presence of *P. Multocida* within domestic animals is a risk to patients who receive scratches or bites. Early wound infection of *P. Multocida* development may cause only erythema and tenderness around the wound site but can develop into serosanguinous or purulent drainage. In rare cases it has been reported to progress to infective endocarditis (IE) if left untreated, escalating the need for early detection, treatment and further diagnostic testing such as transthoracic echocardiogram to exclude IE.

## Introduction

The prevalence of surgical site infections (SSIs) was reported in the region of 11.4% in patients undergoing lobectomy within a European study. However, ‘Getting it Right First Time’ (2019) report in the first national survey of SSIs within the United Kingdom (UK) to categorise thoracic SSIs at 3.7% [1]. There is some discrepancy within the reporting of SSIs within the UK as not all SSIs fall under mandatory surveillance reporting and this can be demonstrated within the reporting period of 2023 to 2024 with no provider reporting on SSIs within thoracic surgery [2]. The SSI surveillance service does not require thoracic surgical procedures to be reported under the mandatory or voluntary surgical procedures carried out in the UK [2]. With the lack of a mandatory requirement, coupled with similar experiences within cardiothoracic centers in the UK where SSI reporting is heterogenous and inconsistent due to a plethora of barriers. It is difficult for clinical staff to collate best evidence practice to share for benchmarking on the identification and management of SSIs not commonly seen such as *Pasteurella multocida (P. Multocida)* [3].

## Case report

A 77-year-old female patient underwent uniportal video-assisted thorascopic surgery for a diagnostic wedge frozen section of the right upper and lower lobe, with right upper lobe completion for a confirmed diagnosis of synchronous adenocarcinoma. The procedure was unremarkable, and the right middle and lower lobes fully expanded, and the patient was extubated.

**Figure 1:**
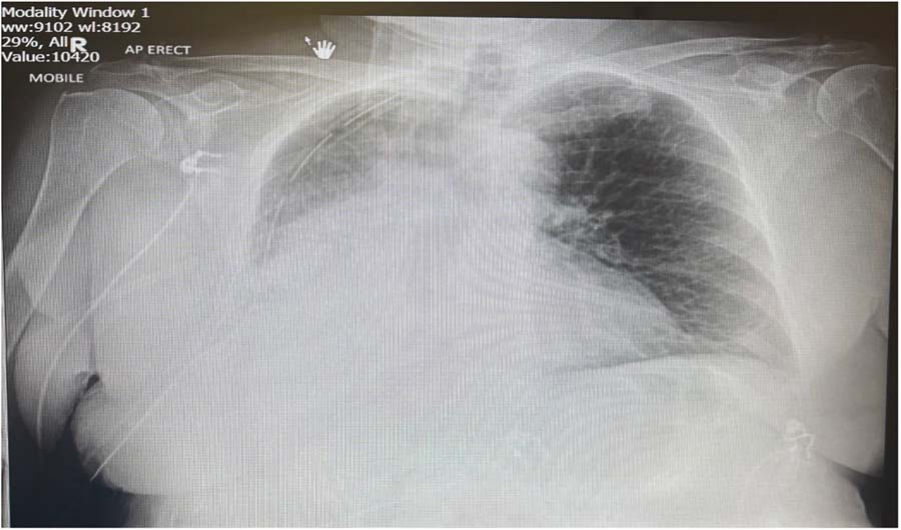
Portable chest X-ray Day 2 postoperatively. Chest X-ray depicts right middle and lower zone consolidation with associated pleural effusion.

**Figure 2:**
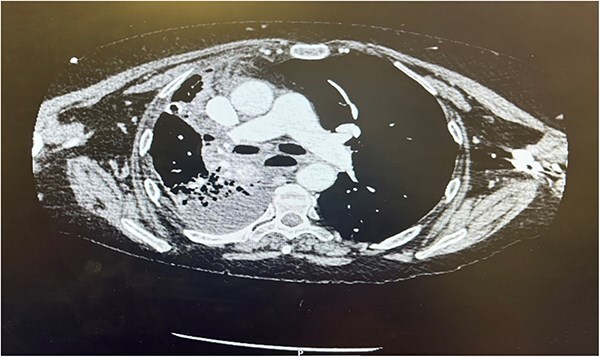
CT thorax Day 3 postoperatively. Right sided pleural effusion with overlying atelectasis and segmental collapse of the right middle and lower lobe.

Postoperatively the patient required supplemental oxygen of 4 liters per minute that escalated to high flow nasal canula with 60 liters per minute. A chest x-ray depicted a right sided collection and collapse (Fig. 1). A computed tomography (CT) of the thorax was requested due to concerns from the chest x-ray and increased oxygen demand to maintain patient oxygen saturations between 88–92%. The CT confirmed a right sided moderate collection with secondary collapse and atelectasis of the lung suggestive of postoperative hemorrhagic changes (Fig. 2).

**Figure 3:**
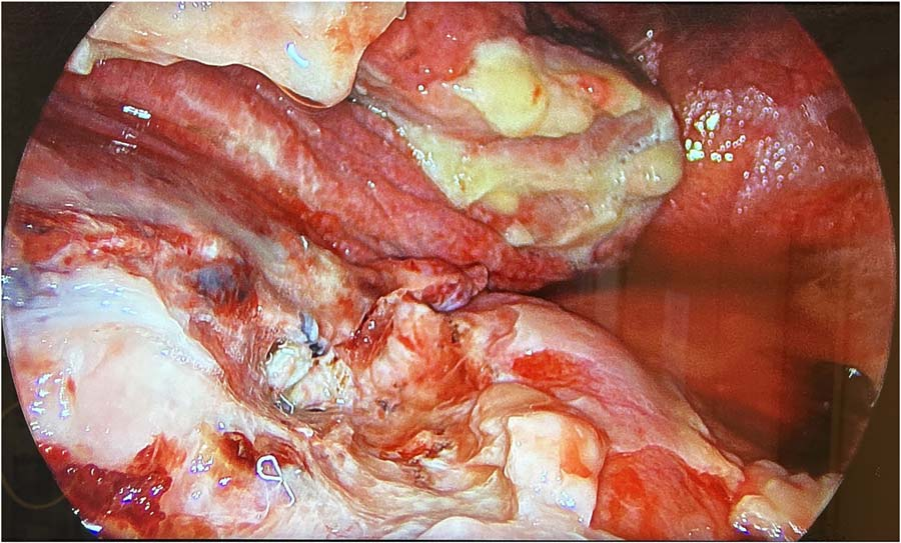
UVATS camera image depicting fibrin tissue formation of early stage empyema within the hilar and azygos region.

Therefore, the patient was consented for a washout of the chest, and this was conducted on Day 3 from the original procedure. Upon establishing access, it was clear that the collection depicted on the CT thorax was not hemorrhagic but mucopurulent fluid in the presence of an empyema (Fig. 3). Due to the mucopurulent fluid and presence of early-stage organisation of septations and loculated fluid, the empyema was fibro purulent. In line with the European Association for Cardio-Thoracic Surgery (EACTS) consensus and British Thoracic Society (BTS) guidelines for the management of pleural disease, the empyema was graded as stage 2 [4–6].

Decortication of the fibrin depositions on the parietal and visceral pleura was carried out with samples taken for microbiology. The patient was irrigated with saline wash and two drains inserted into the apical and basal regions.

## Discussion

Pleural infection such as empyema remains a common condition with significant mortality of 20% within 12 months of original diagnosis and requires 15% of patients to be treated surgically [4]. The incidence of empyema has seen a steady rise from 4447 cases in 2008 to 7268 in 2017 [5]. Empyema development is more commonly associated with bacterial pneumonia, chest wall injury, tuberculosis, or infection after thoracic surgery [4, 5, 7]. With the early development of empyema in this case presentation having undergone thoracic surgery, and the absence of any animal scratches or bites leaves the mode of transmission unknown. The mode of transmission from domestic animal to human infection is passed commonly through scratches, bites, licking, or the inhalation of infected secretions. While the patient did confirm they had a cat, there was no evidence of any recent bites or scratches on the upper or lower limbs on inspection.

The incidence of Pasteurella infections within the UK reported 801 cases in 2022, 815 cases in 2023, and 219 cases within quarter 1 of 2024 [8]. However, this figure is likely to be higher as not all cases are reported with hospital admission and less complex cases treated locally with primary care input [8]. While the incidence is relatively low, the complications arising from a gram-negative bacterium presents a risk of infective endocarditis (IE) that is normally associated with gram-positive bacterium but carries a mortality rate of 17.9% with *P. Multocida* infection [9]. The incidence of valve involvement with IE occurred more commonly involving the aortic valve (50%), mitral valve (30.8%) and less commonly multiple valves (3.8%) [9]. Therefore, the need to exclude IE by transthoracic echocardiogram (TTE) is a significant diagnostic examination to exclude an infection that can take 8 to 14 days from initial transmission [9]. A TTE was undertaken and concluded the absence of any IE. Concurrently the antibiotic regime was changed to Doxycycline 100 mg once per day for 7 days in line with the British National Formulary [10]. However, the length of treatment was extended to 4 weeks as this is supported by the BTS guidelines advising antibiotic regimes to cover a period of 2 to 6 weeks to reduce the chance of clinical relapse [9, 10].

## Conclusion

With significant risk of mortality from empyema and need for early surgical intervention, it is imperative for early detection and management of pleural disease in line with current BTS guidelines. Empyema is commonly associated with arising from bacterial pneumonia, chest wall injury, tuberculosis and thoracic surgery, but has been the result in this case study of a less common infection of zoonotic origin. Furthermore, IE and the less common association with gram negative bacteria is a consideration to exclude within patients presenting with this type of infection with the diagnostic imaging to include TTE.
